# Isolation of Antiosteoporotic Compounds from Seeds of *Sophora japonica*

**DOI:** 10.1371/journal.pone.0098559

**Published:** 2014-06-03

**Authors:** Hossam M. Abdallah, Ahmed M. Al-Abd, Gihan F. Asaad, Ashraf B. Abdel-Naim, Ali M. El-halawany

**Affiliations:** 1 Department of Natural Products, Faculty of Pharmacy, King Abdulaziz University, Jeddah, Saudi Arabia; 2 Department of Pharmacognosy, Faculty of Pharmacy, Cairo University, Cairo, Egypt; 3 Pharmacology Department, Medical Division, National Research Center, Giza, Egypt; 4 Department of Pharmacology and Toxicology, Faculty of Pharmacy, King Abdulaziz University, Jeddah, Saudi Arabia; 5 Pharmacology Department, Faculty of Pharmacy, Jazan University, Jazan, Saudia Arabia; Clermont Université, France

## Abstract

Chemical investigation of *Sophora japonica* seeds resulted in the isolation of seven metabolites identified as: genistin (**1**), sophoricoside (**2**), sophorabioside (**3**), sophoraflavonoloside (**4**), genistein 7,4’-di-*O*-*β*-D-glucopyransoide (**5**), kaempferol 3-*O*-*α*–L-rhamnopyranosyl(1→6)*β*-D-glucopyranosyl(1→2)*β*-D-glucopyranoside (**6**) and rutin (**7**). Compounds **1**, **2** and **5** showed significant estrogenic proliferative effect in MCF-7 cell in sub-cytotoxic concentration range. Compounds **1** and **2** showed minimal cell membrane damaging effect using LDH leakage assay. Accordingly, compound **2** (sophoricoside, (SPH)) was selected for further *in-vivo* studies as a potential anti-osteoporosis agent. The anti-osteoporotic effect of SPH was assessed in ovarectomized (OVX) rats after oral administration (15 mg/kg and 30 mg/kg) for 45 days compared to estradiol (10 µg/kg) as a positive control. Only in a dose of 30 mg/kg, SPH regained the original mechanical bone hardness compared to normal non-osteoporotic group. However, SPH (15 mg/kg) significantly increased the level of alkaline phosphatase (ALP) to normal level. Treatment with SPH (30 mg/kg) increased the level of ALP to be higher than normal group. SPH (15 mg/kg) did not significantly increase the serum level of osteocalcin (OC) compared to OVX group. On the other hand, treatment with SPH (30 mg/kg) significantly increased the level of OC to 78% higher than normal non-ovarectomized animals group. In addition, SPH (15 mg/kg) decreased the bone resorption marker, acid phosphatase (ACP) to normal level and SPH (30 mg/kg) further diminished the level of serum ACP. Histopathologically, sophoricoside ameliorated the ovarectomy induced osteoporosis in a dose dependent manner. The drug showed thicker bony trabeculae, more osteoid, and more osteoblastic rimming compared to OVX group.

## Introduction

According to the World Health Organization “Osteoporosis is a disease characterized by low bone mass and micro-architectural deterioration of bone tissues, leading to enhanced fragility and consequent increase in fracture risk that results in fractures with minimal trauma”. Osteoporosis, a silent epidemic has become a chief health hazard in recent years, afflicting over 2000 million people worldwide [1]. Osteoporosis is associated with deficiency of ovarian hormone following menopause. A sharp decrease in ovarian estrogen production is the predominant cause of rapid, hormone-related bone loss after menopause [2] as a result of higher bone turnover, an imbalance between bone formation and bone resorption & net bone loss [3]. The common sites of fracture among postmenopausal women include the vertebrae, forearm and hip. The incidence of hip fractures & cost for treatment will rise in the future, unless successful prophylactic actions are taken [4].

In Saudi Arabia, the incidence of osteoporosis is common among postmenopausal women; it is often associated with early or late onset of menopause. It was found that osteoporosis is familiar (60%) among postmenopausal Saudi Arabian women [5], [6].

Hormone replacement therapy (HRT) effectively ameliorates postmenopausal symptoms and lowers the risk for coronary heart disease and osteoporosis. However, HRT increases the risk of breast cancer and cardiovascular diseases. To overcome the wide range of side effects produced by HRT, there is an increasing demand for “backing to nature” which is considered to be healthier and safer for the treatment of osteoporosis. Phytoestrogens are plant-derived compounds that structurally or functionally mimic mammalian estrogens, and therefore are considered to play an important role in the prevention of cancers, heart disease, menopausal symptoms, and osteoporosis.

Recent reports indicate that phytoestrogens exert their effects in selective estrogen receptor modulators (SERMs) -like manner [7]. The plant food sources high in phytoestrogens are numerous and include soybeans, flaxseeds, and certain other fruits and vegetables rich in polyphenolic compounds.

*Sophora japonica* L. family Fabaceae, is a tree native to China and Korea. It is also named Japanese pagoda tree (Enju) or Chinese scholar tree. It has been used in Chinese traditional medicine as a haemostatic agent. Flavones from the buds and pericarp were discovered as haemostatic constituents [8], [9]. Triterpenes, phospholipids, alkaloids, amino acids and fatty acids have been reported as the main chemical constituents of the seeds of this plant [10], [11].

The naringinase-digested methanol extract of *S. japonica* seeds showed potent estrogen agonist activity due to its genistein and kaempferol contents [12]. Genistein from *S. japonica* was also reported to prevent osteoporosis [13]. Moreover, *in-vivo* studies have shown that *S. japonica* extracts prevented bone loss, partly by inhibiting osteoclastic activity [14]. Dichloromethane (DCM) of *S. japonica* fruit extract stimulated alkaline phosphatase activity and matrix mineralization. The DCM fraction also induced expression of osteoblast markers such as alkaline phosphatase, osterix, and osteocalcin in C3H10T1/2 cells and primary bone marrow cells [15].

In the present study the major phenolic compounds from *S. japonica* seed were isolated and their estrogenic activity was determined in MCF-7 cells. Compound that proved highest estrogenic proliferative activity was tested *in-vivo* for its osteoprotective effect in ovarectomized rats.

## Results

**Figure 1:**
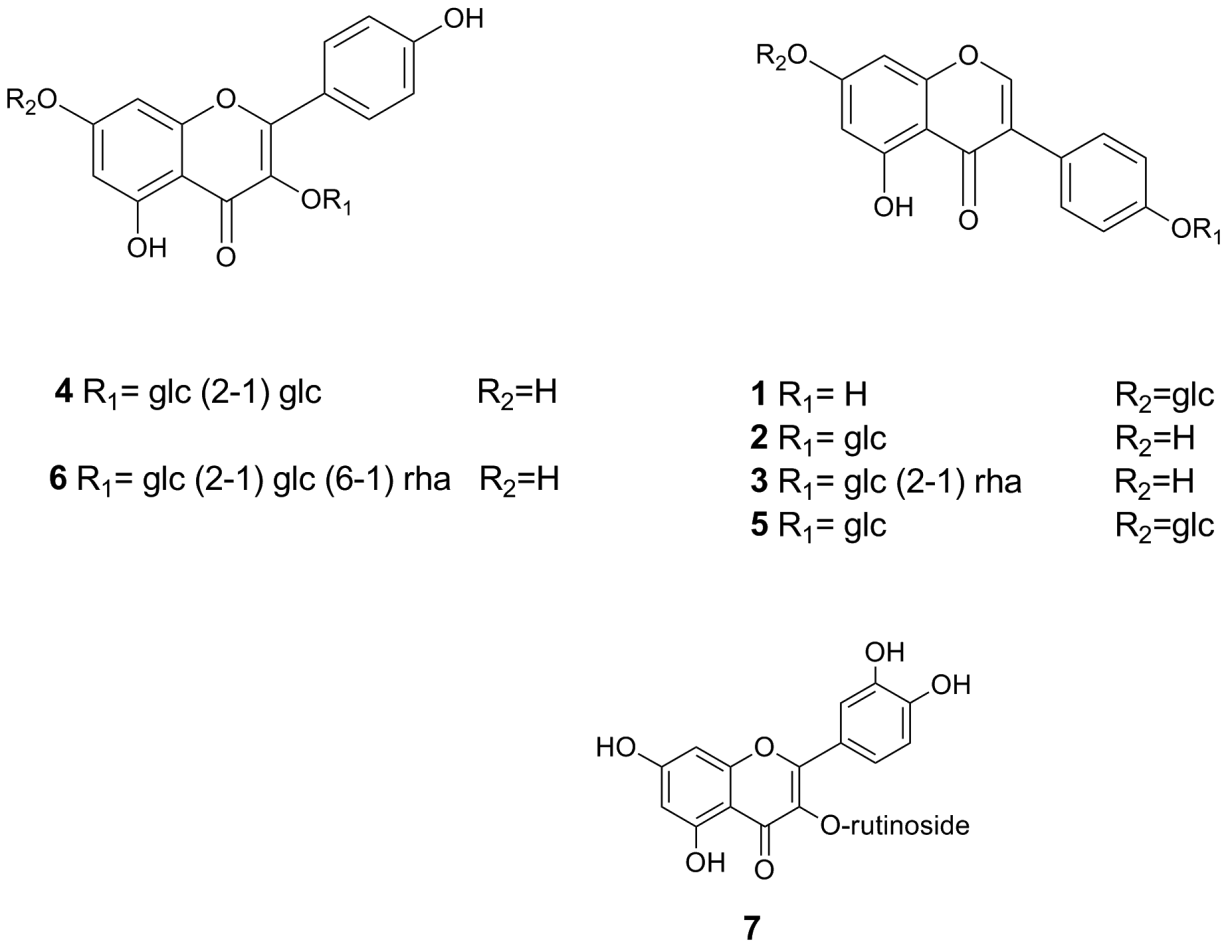
Chemical structure for compounds isolated from *Sophora japonica* seeds.

The methanol extract of *S. japonica* seeds was partitioned with chloroform and the remaining water soluble portion was fractionated on a Diaion HP-20 column to afford 25%, 50%, and 100% methanol fractions. The 50% methanol fraction was purified on several silica gel, ODS and Sephadex LH-20 columns to produce seven compounds. The identification of isolated compounds was achieved systematically by trying the response of the isolated compounds to different spray reagents using thin layer chromatography (TLC) aiming to identify the nature of the compounds. ^1^H NMR study was carried out for all compounds to, un-ambiguously, identify its structure. Compounds with more than one sugar moiety such as compounds **3–6** were further subjected to ^13^C NMR measurement to clearly identify site of sugar attachment (Figure S1–S5). The identity of compounds was confirmed by comparing NMR data with previously reported ones as (Figure S1–S5, Table S1), genistin (**1**) [16], sophoricoside (**2**) [17], sophorabioside (**3**), sophoraflavonoloside (**4**), genistein 7,4’-di-*O*-*β*-D-glucopyransoide (**5**) [18], kaempferol 3-*O*-*α*–L-rhamnopyranosyl(1→6)*β*-D-glucopyranosyl(1→2)*β*-D-glucopyranoside (**6**) [19], and rutin (**7**) (Fig. 1). Purity of all isolated compounds was confirmed through HPLC analysis. The purity of compounds **4–6** was over 92%, while that of the remaining compounds was over 95% as indicated by their HPLC chromatograms (Figure S6).

### The estrogenic/proliferative properties of compounds isolated from *S. japonica* in MCF-7 cell line

The proliferative effect in estrogen dependent MCF-7 cell line of the isolated compounds was tested. Sub-cytotoxic dose range with potential estrogenic proliferative effect of compounds under investigation was determined using trypan blue exclusion assay and further confirmed using LDH leakage assay.

Trypan blue positive cells was less than 50% after exposure to all compounds under investigation indicative of negative cytotoxic effect until 100 µM concentration. Exposure to 1 mM of compounds **3**, **4**, **6**, and **7** induced trypan staining in more than 50% of cells. On the other hand, exposure to compounds **1**, **2**, and **5** showed trypan blue positive cells less than 1% until 100 µM concentration; and accordingly these compounds can be considered the safest to cell integrity (Table 1).

**Table 1:** Cytotoxicity assessment of compounds isolated from *S. Japonica* using trypan blue exclusion assay.

Cpd	Compound name	Percent dead cells				
		0.1 µM	1 µM	10 µM	100 µM	1 mM
1	Genistin	<1%	<1%	<1%	<1%	42.5±3.6%
2	Sophoricoside	<1%	<1%	<1%	<1%	39.2±2.8%
3	Sophorabicoside	<1%	<1%	2.4±0.2%	15.7±2.7%	63.9±5.1%
4	Sophoraflavonoloside	<1%	1.5±0.3%	8.3±2.6%	17.5±3.1%	74.2±7.4%
5	genistein glucoside	<1%	<1%	<1%	<1%	41.7±3.7%
6	kaempferol glucoside	2.6±0.8%	8.6±1.5%	14.8±3.6%	21.7±4.2%	87.3±5.1%
7	Rutin	<1%	<1%	6.8±2.15	14.3±3.1%	68.5±4.9%

**Figure 2:**
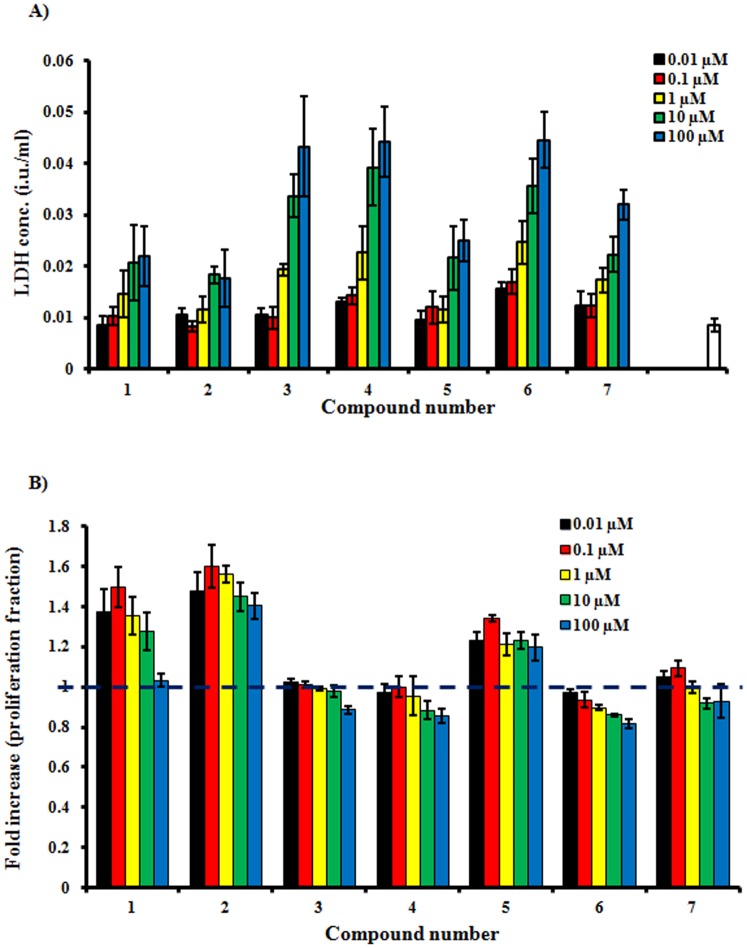
Assessing the estrogenic properties of compounds isolated from *S. japonica* in estrogen dependent MCF-7 cell line. Sub-cytotoxic concentrations of the isolated compounds were determined using LDH leakage assay (A); and proliferative effect was determined using SRB assay (B).

Effect of compounds under investigation on the cell membrane integrity was further confirmed using more sensitive technique (LDH leakage assay). Exposure to 1 µM of compounds **3**, **4** and **6** induced 2.2, 2.6 and 2.8 folds LDH leakage that in the control group, respectively. The concentration of LDH in media was about 5 folds control group after exposure to 100 µM of compounds **3**, **4** and **6**. On the other hand, the amount of LDH in media over cells treated with concentrations less than 10 µM of compounds **1**, **2**, **5**, and **7** did not exceed double the concentration of LDH leakage in control group. These indicate the harmless effect of these compounds on cell membrane (Figure 2-A).

With respect to the proliferative effect in MCF-7 cell, compounds **3**, **4**, and **6** did not show any significant proliferation enhancement after 72 h of exposure to concentration range of 0.01 to 100 µM. The proliferative effect of compounds **1**, **2**, and **5** was significant in MCF-7 starting from a concentration of 0.01 µM (1.4, 1.5, and 1.2 folds, respectively); and most prominent at concentration of 0.1 µM showing 1.5, 1.6, and 1.3 folds, respectively (Figure 2-B). Based on the cytotoxicity profile, effect on membrane integrity and estrogenic proliferative activity; compound **2** (sophoricoside) was selected for further studies as a potential anti-osteoporosis agent *in-vivo*.

### Anti-osteoporosis effect of sophoricoside *in-vivo*

The anti-osteoporosis effect of sophoricoside was investigated mechanically and biochemically in ovarectomized rats after oral administration (15 mg/kg and 30 mg/kg) for 45 days compared to estradiol (10 µg/kg). Mechanical hardness of femur bones was determined after applying longitudinal and vertical forces to the bone shaft and identifying the breaking point pressure.

**Figure 3:**
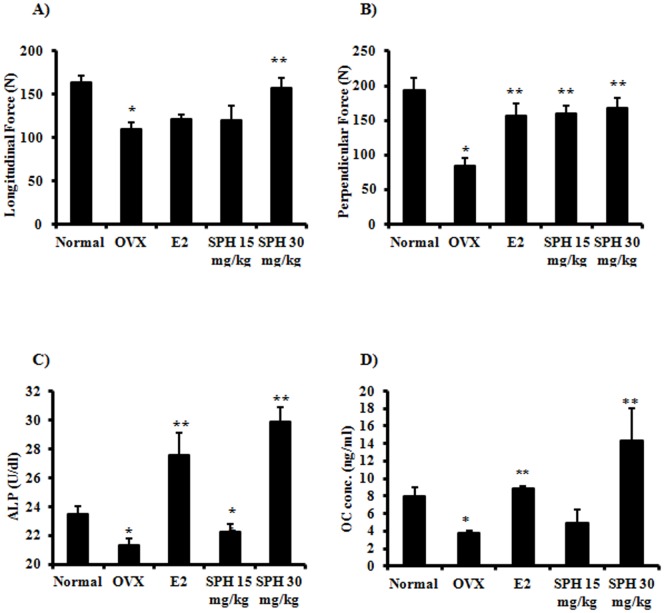
Mechanical and biochemical assessment for anti-osteoporosis effect of sophoricoside *in-vivo.* Ovariectomized rats were treated with SPH (15 mg/kg and 30 mg/kg) for 6 weeks and compared to E_2_ treated animals (10 µg/kg) and sham-operation group. Mechanical hardness was evaluated using hardness tester along (A) and perpendicular (B) to femur bone shaft. Biochemical assessment of osteoporosis was evaluated by measuring serum alkaline phosphatase (C) and osteocalcin (D) at the end of treatment period.

Mechanically, the forces required to break the femur bones longitudinally and vertically were significantly decreased in response to ovarectomy to be 34% and 56% of the normal bone hardness, respectively. Treatment with E_2_ (10 µg/kg) and low dose SPH (15 mg/kg) did not affect the bone hardness in response to longitudinal force; however high dose SPH (30 mg/kg) improved the bone hardness by 43.3% compared to OVX group. In other words, treatment with SPH (30 mg/kg) regained the original longitudinal bone hardness (Figure 3-A). On the other hand, treatment with E_2_ (10 µg/kg), SPH (15 mg/g) and SPH (30 mg/kg) significantly improved bone hardness to vertical force by 89.3% and 97.4%, respectively compared to OVX group (Figure 3-B).

Osteoporosis was assessed biochemically by measuring the serum concentration of alkaline phosphatase (ALP) and osteocalcin (OC) as markers for bone formation. In addition, acid phosphatase (ACP) was measured as an indicator for bone resorption. Serum level of ALP was significantly decreased in OVX group indicative of osteoporosis. Treatment with E_2_ significantly increased the level of ALP to above normal level. Treatment with sophoricoside (15 mg/kg) significantly increased the level of ALP and brought it to normal level. However, treatment with sophoricoside (30 mg/kg) increased the level of ALP to be higher than normal group (Figure 3- C).

Similar to ALP, Serum level of OC was significantly decreased to about half its original value in response to ovarectomy confirming the incidence of osteoporosis. Treatment with E_2_ significantly increased the level of OC back to its normal level. Treatment with sophoricoside (15 mg/kg) did not significantly increase the serum level of OC compared to OVX group. However, treatment with sophoricoside (30 mg/kg) significantly increased the level of OC to 78% higher than normal group non-ovarectomized animals (Figure 3- D).

Serum level of ACP was significantly increased (60% compared to control group) in response to OVX indicating prominent hormone dependent bone resorption. ACP level was significantly depressed by E_2_ treatment to reach 17.2% of OVX group level. Treatment with sophoricoside (15 mg/kg) significantly decreased the level of ACP and brought it to normal level. Surprisingly, treatment with sophoricoside (30 mg/kg) extensively decreased the level of ACP to be only 4.5% of OVX group level (Figure 3- E).

### Histopathological assessment of the anti-osteoporosis effect of sophoricoside

**Figure 4:**
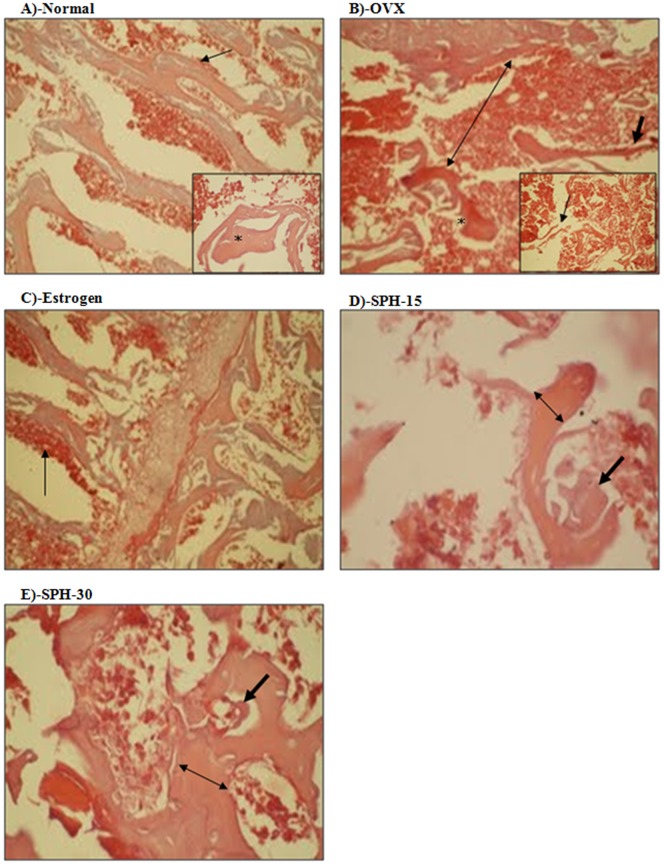
Histopathological assessment of the anti-osteoporosis effect of sophoricoside. Normal group (A) shows normal bony tissue with intact well-formed dense trabeculae (star in the inner panel) with osteoblastic rimming (arrow) and average intervening bone marrow. OVX group (B) showed scant, disconnected (arrow in inner panel), thin (arrow in the outer panel), and widely separated trabeculae (double-headed arrow in the outer panel) with eroded surface (star in the outer panel). Estrogen treated group (C) showed widely distributed osteoid and osteoblastic rimming (arrow). SPH-15 group (D) showed thick trabeculae (double-headed arrow), more osteoid, and osteoblastic activity (arrow). SPH-30 group (E) showed thick trabeculae (double-headed arrow), more osteoid and osteoblastic activity (arrow).

The anti-osteoporotic effect of sophoricoside was confirmed pathologically. Histological pictures of femurs of normal animals showed intact, well-formed, dense bony trabeculae with osteoblastic rimming and average intervening bone marrow (Fig. 4-A). Ovarectomy resulted in prominent osteoporosis in OVX group manifested as thin and widely separated trabeculae with notched and eroded surfaces (Fig. 4-B). Estradiol partly reversed the osteoporosis status showing thicker bony trabeculae and more osteoid and osteoblastic rimming compared to OVX group (Fig. 4-C). Sophoricoside ameliorated the ovarectomy induced osteoporosis in a dose dependent manner. Femurs of animals treated with SPH (15 mg/kg) showed thicker bony trabeculae, more osteoid, and more osteoblastic rimming compared to OVX group (Fig. 4-D). Femurs of animals treated with higher dose of SPH (30 mg/kg) showed thicker bony trabeculae, higher osteoid activity with narrow bone marrow spaces compared to OVX group (Fig. 4-E).

## Discussion

Osteoporosis is a major health problem particularly for post-menopause women due to hormonal deficiency [15]. The osteo-protective use of natural compounds with estrogenic activity enabled the avoidance of estrogen replacement therapy and its debilitating side effects [15]. Natural products with inherit nutritional value on top of estrogenic activity such as *S. japonica* represent an interesting nutraceutical alternative for osteoporosis [20]–[22]. In the current study, we examined the estrogenic proliferative activity of seven compounds isolated from *S. Japonica* in the estrogen dependent MCF-7 cell line. The best candidate estrogenic compound (Sophoricoside) was further tested for potential osteo-protective activity using ovarectomized rat model.

In the current study, genistin, sophoricoside and genistein 7,4’-di-*O*-*β*-D-glucopyransoide showed significant proliferative activity in estrogen dependent MCF-7 cell line in a subcytotoxic concentration range. Sophoricoside which is highly abundant phytoestrogen in the methanolic extract of *S. Japonica* seeds [12] did not induce any tangible cell membrane damage up to 100 µM concentration. In a previous report, prominent anti-osteoporosis effect of dichloromethane extract of *S. Japonica* fruits was attributed to the high content of the genistein aglycone as evidenced by LC-MS analysis [15]. Sophoricoside which is another genistein glycoside, possess potent estrogenic activity as assessed in our previous work using yeast hybrid assay [12].

Herein, sophoricoside (15 mg/kg and 30 mg/kg) showed promising and dose dependent osteo-protective effect against ovarectomy induced osteoporosis rat model compared to estradiol. Sophoricoside improved the mechanical bone hardness in ovarectomized rats after treatment for 6 weeks. Sophoricoside (4–16 mg/kg for 4 weeks) improved the pathological picture of trabecular bones in ovarectomized rats in previous report [20]; however, herein both mechanical as well as pathological evidences are presented for the osteo-protective effect of sophoricoside. In addition, it was observed that sophoricoside induced significant elevation in the osteogenic biochemical markers such as serum alkaline phosphate and osteocalcin.

Osteo-protective effect of sophoricoside might be attributed to the resulting genistein after hydrolysis in GIT flora or directly to the parent compound *per se*. Therefore, the use of the total alcoholic extract of *S. japonica* seeds could be as effective as sophoricoside due to its high content of geniestein-derived glycosides which will be transformed to genistein upon hydrolysis by GIT flora.

## Materials and Methods

### General

TLC was carried out on precoated Silica gel 60 F_254_ (0.25 mm, Merck) and RP-18 F_254_S (0.25 mm, Merck Co., Dermstadt) and spots were detected under UV light or after spraying with anisaldehyde-H_2_SO_4_ reagent followed by heating. Column chromatography (CC) was carried out on (BW-820MH silica gel), Wakosil C-300 (40–64 *µ*m) (Wako, Osaka, Japan), ODS DM 1020T (ODS, Fuji Silysia, Nagoya, Japan), Diaion HP-20 (Mitsubishi Kasei, Tokyo, Japan) and Sephadex LH-20 (Pharmacia Co.). Medium pressure liquid chromatography (MPLC) was performed on LiChroprep RP-18 and LiChroprep Si 60 (size A and B, Merck Co.). HPLC analysis was conducted on Agilent 1200 liquid chromatography equipped a photodiode array detector. A C18 reversed-phase packing column (4.5 mm×15 cm, 5 µm) were used for separation throughout this study.

### Plant material

Seeds of *S. japonica* were collected from the ripe fruits cultivated in the Medicinal Plant Station of Faculty of Pharmacy, Cairo University during December 2012. Authentication of the plant was established by Ass. Prof. Dr. Sherif El-Khanagry, Agriculture Museum, El-Dokki, Cairo, Egypt. A Herbarium specimen (SJ-1023) was prepared and kept at the Herbarium of the Department of Natural Products and Alternative Medicine, Faculty of Pharmacy, King Abdulaziz University.

### Extraction and isolation

The pulverized seeds of *S. japonica* (1300 g) were extracted with MeOH (1L x 3) at room temperature and the combined extract was evaporated in *vacuo*. The methanol extract (104 g) was suspended in MeOH and water and successively partitioned with hexane and chloroform to produce hexane (31 g) and chloroform (4 g) soluble fractions. The remaining aqueous layer was fractionated on a Diaion HP-20 column (60 cm×6 cm) stepwisely eluted with H_2_O, 25%, 50% and 100% MeOH. The eluates were evaporated under vacuum to afford H_2_O fraction (26 g), 25% MeOH fraction (8.5 g), 50% MeOH fraction (14 g) and 100% MeOH fraction 5.8 g. The 50% MeOH (12 g) was applied to a silica gel column (300 g silica) and gradienltly eluted with CHCl_3_-MeOH-H_2_O (9∶1∶0.1 v/v/v) to obtain 8 fractions. Fraction 3 (2 g) gave a yellow precipitate upon concentration, that was washed several times by chloroform-methanol to obtain compound **3** (193 mg). The supernatant of this fraction was purified on an MPLC RP-18 column (size A) using MeOH-H_2_O (4∶6 v/v) to afford compounds **1** (120 mg), and **2** (500 mg). Fraction 4 gave compound **4** (358 mg) upon crystallization from MeOH and the remaining supernatant was applied to an MPLC RP-18 column (size A) eluted with MeOH-H_2_O (3∶7 v/v) to get **7** (5 mg). Fraction 5 gave compound **5** (180 mg) by crystallization from MeOH-CHCl_3_.Fraction 6 (1.5 g) was purified on a Sephadex LH-20 column (30 cm×3 cm) eluted with MeOH-H_2_O (1∶1 v/v). Sub-fractions 17–30 of this column was combined together and applied to an MPLC RP-18 column (size A) eluted with MeOH-H_2_O (3∶7 v/v) to get compound **6** (37.4 mg). Purity of all compounds were analyzed using HPLC using a gradient elution (water – TFA, 0.1% v/v) to solvent B (acetonitrile), with a flow rate of 1.0 ml min

### Chemicals and drugs

Sulfarhodamine B (SRB) and 17β-hydroxyesradiol were purchased from Sigma-Aldrich Chemical Company (St. Louis, MO, USA), RPMI-1640 media, fetal bovine serum and other cell culture materials were purchased from Euroclone (Milano, Italy). Urethane was purchased from Biobasic Inc. (Toronto, Canada). All other chemicals were of the highest available analytical grade.

### Cell culture

Human estrogen dependent breast adenocarcinoma cells (MCF-7 cell line) were obtained from the Vacsera (Giza, Egypt). Cells were maintained in RPMI-1640 supplemented with 100 µg/mL streptomycin, 100 units/mL penicillin and 10% heat-inactivated fetal bovine serum in a humidified, 5% (v/v) CO_2_ atmosphere at 37°C.

### Cytotoxicity assessment

Cytotoxicity of compounds isolated from *S. Japonica* was tested in MCF-7 cells by trypan-blue exclusion assay. Briefly, exponentially growing cells were plated in 96-well plates. Cells were exposed to serial dilutions of isolated compounds for 2, 4, 7 and 10 days and the percent of trypan blue positive cells was determined in live culture [1], [23].

### Cell membrane integrity assessment

The influence of compounds isolated from *S. Japonica* against the cell membrane integrity was assessed in MCF-7 cells by LDH leakage assay [24]. Briefly, exponentially growing cells were plated in 6-well plates. Cells were exposed to serial concentrations of isolated compounds for 24 h and the LDH was determined in the culture media using colorimetric assay (Biosystems, Barcelona, Spain).

### Proliferation assay

The proliferative effect of compounds isolated from *S. Japonica* was tested in MCF-7 cells by SRB assay as well. Briefly, exponentially growing cells were exposed to sub-cytotoxic concentrations of the isolated compounds for 72 h and subsequently stained with SRB solution for quantification [25], [26].

### Animals and animal treatment

Animal handling and treatment was approved by the bioethical and research committee of The National Research Center. Female Sprague Drawly rats (300 g weight) were acclimatized in the animal house facility of The National Research Center, Cairo, Egypt, for at least one week prior to experimentation. Animals were kept at 20±2°C and 65±10% relative humidity during the whole experiment. Standard food pellets and water were supplied *ad labium*. All experiments were performed between 8–10 A.M.

Osteoporosis was induced in rats by ovarectomy as previously described with minor modification [3]. Briefly, rats in the same menstrual phase were selected and confirmed by vaginal smear and underwent experimental ovarectomy. Animals were anesthetized by i.p. injection of urethane (1 g/kg). Surgical incision of about 5 mm was made in each of the hind dorsal flank regions of rats exposing the ovary and surrounding periovarian fat bed. Both fallopian tubes were suture-closed at the most distal end and both ovaries were surgically excised carefully. The incision was sutured and dressed with sterile povidone iodine solution. Sham operations were performed to animals of control groups. Animal were let for two weeks to recover from the surgical trauma. Then, animals were divided into 5 groups (n = 10 per group). Control group which was subjected to sham operation; OVX group was surgically ovarectomized and received saline vehicle daily. Estrogen group (E_2_) was subjected to surgical ovarectomy and received 17β-estradiol (10 µg/kg) via i.p. injection daily; sophoricoside low dose group (SPH-15) was subjected to surgical ovarectomy and received sophoricoside (15 mg/kg) p.o. daily. Sophoricoside high dose group (SPH-30) was subjected to surgical ovarectomy and received sophoricoside (30 mg/kg) p.o. daily. All animals were allowed free access for food and water throughout the whole experiment (45 day). Blood samples were withdrawn by retro-orbital plexus puncture; and the serum was collected then animals were sacrificed by cervical dislocation. Both right and left femur bones were dissected immediately *post mortem*; left femurs were used to measure hardness; and the right femurs were fixed in buffered formalin solution (4%) for histological assessment.

### Bone hardness assessment

To quantify the degree of osteoporosis, hardness of left femurs was measured using hardness tester (Erweka GmbH, Heusenstamm, Germany) as previously described with minor modification [27]. Briefly, left femurs were placed in the clamp assembly of the hardness tester in a vertical (n = 5) and horizontal (n = 5) positions to the force direction. Minimum force required to induce bone shaft fracture was recorded.

### Serological evaluation of osteoporosis in-vivo

To assess the ossification process in response to treatment with sophoricoside, serum level of alkaline phosphatase (ALP), acid phophatase (ACP) and osteocalcin (OC) were measured. ALP and tartarate-resistant ACP were determined by direct HTS-ready colorimetric assay (Abcam, Cambridge, UK) [28]. Osteocalcin was determined using Uscan immunoassay ELISA Kit (Life Science Inc. Wuhan, China) according to the manufacturer's instructions [29].

### Histological examination for rat lamellar bone tissue

Histological assessment for lamellar bone was performed according the lab routine protocol. Briefly, paraformaldhyde fixed tissues were decalcified by EDTA and embedded in paraffin wax. Cross vertical sections (5 µm) were obtained and after dewaxing and rehydration sections were stained with H&E.

### Statistical analysis

Data are presented as mean ± SEM. Analysis of variance (ANOVA) with LSD post hoc test was used for testing the significance using SPSS for windows, version 17.0.0. *p*<0.05 was taken as a cut off value for significance.

## Supporting Information

Figure S1 **^1^H-NMR charts of compounds 1, 2 and 7.** (A) Compound 1; (B) Compound 2; (C) Compound 7.
(TIF)

Figure S2 **^1^H-NMR and ^13^C-NMR charts of compound 3.**
(TIF)

Figure S3 **^1^H-NMR and ^13^C-NMR charts of compound 4.**
(TIF)

Figure S4 **^1^H-NMR and ^13^C-NMR charts of compound 5.**
(TIF)

Figure S5 **^1^H-NMR and ^13^C-NMR charts of compound 6.**
(TIF)

Figure S6 **HPLC chromatograms of isolated compounds.**
(TIF)

Table S1 **^13^C NMR data of compounds 3-6.**
(DOCX)
