# Supplementary figures and images for: Isolation of Antiosteoporotic Compounds from Seeds of Sophora japonica
Source: PLoS One. 2014 Jun 3;9(6):e98559. doi: 10.1371/journal.pone.0098559 (PMC4043785; doi:10.1371/journal.pone.0098559)

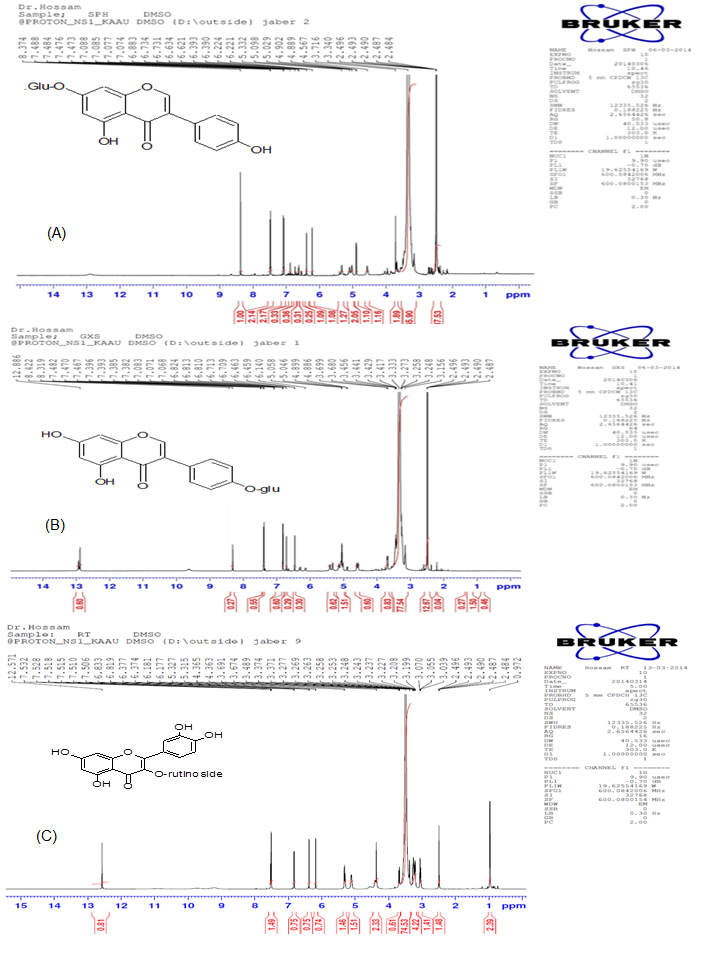

Supplement: Figure S1 — 1H-NMR charts of compounds 1, 2 and 7. (A) Compound 1; (B) Compound 2; (C) Compound 7. (TIF) [file pone.0098559.s001.tif]

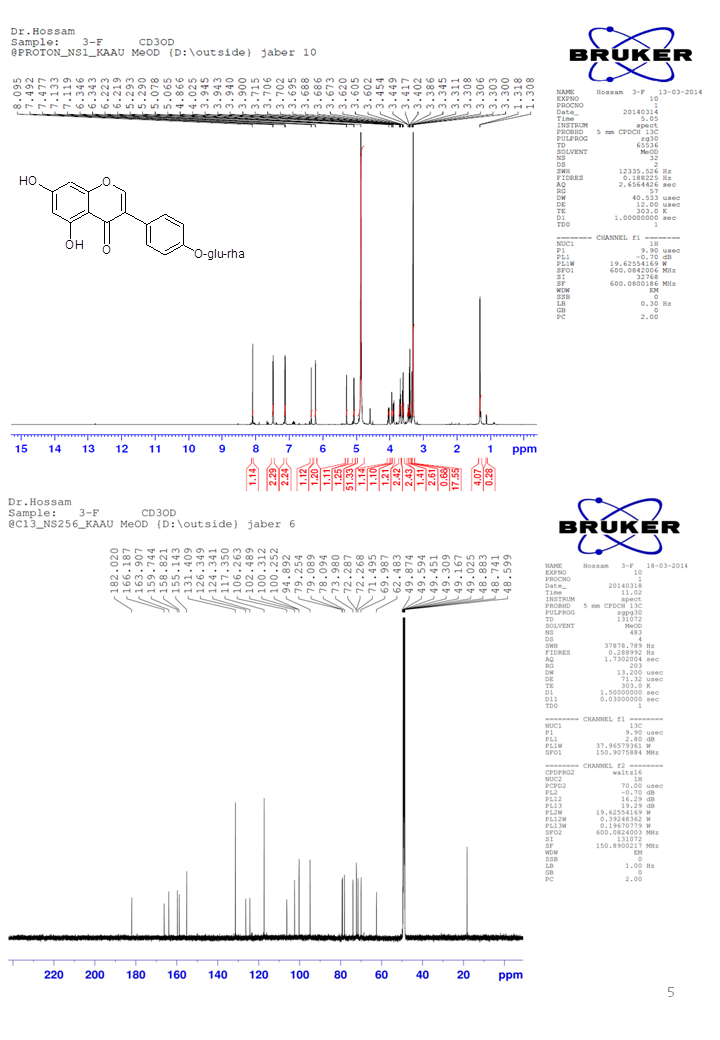

Supplement: Figure S2 — 1H-NMR and 13C-NMR charts of compound 3. (TIF) [file pone.0098559.s002.tif]

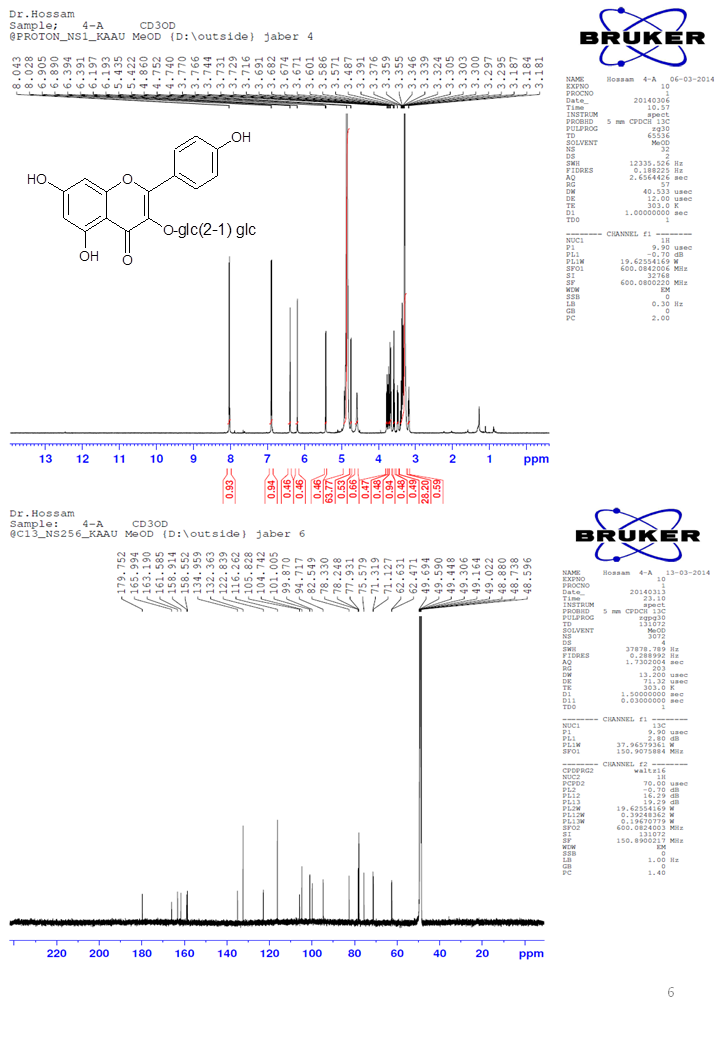

Supplement: Figure S3 — 1H-NMR and 13C-NMR charts of compound 4. (TIF) [file pone.0098559.s003.tif]

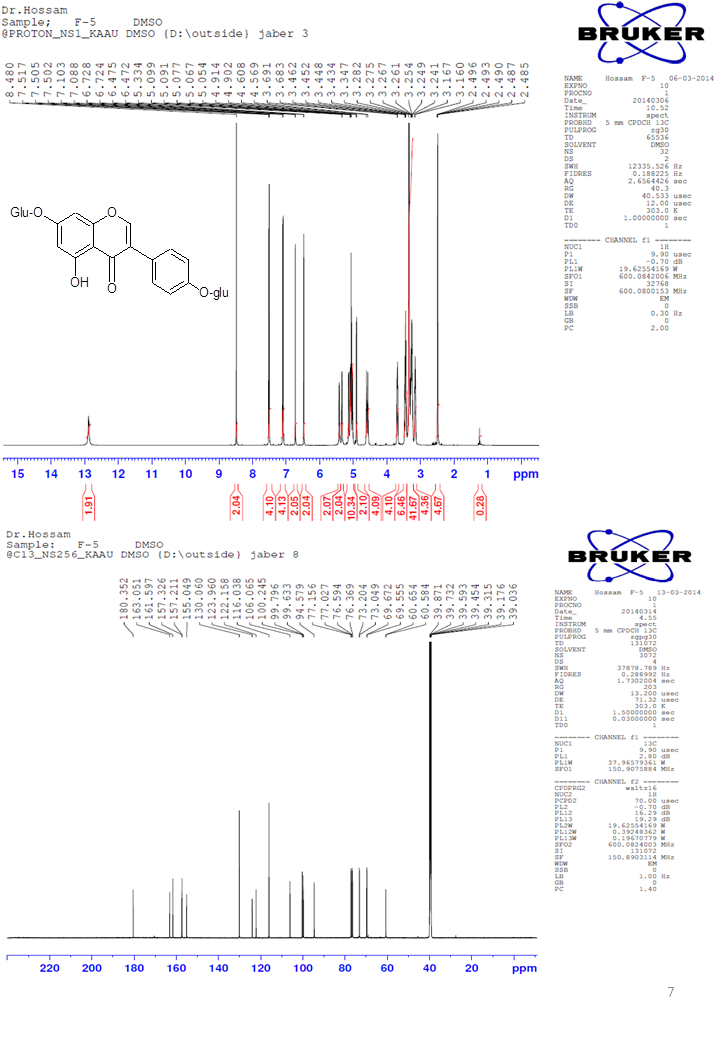

Supplement: Figure S4 — 1H-NMR and 13C-NMR charts of compound 5. (TIF) [file pone.0098559.s004.tif]

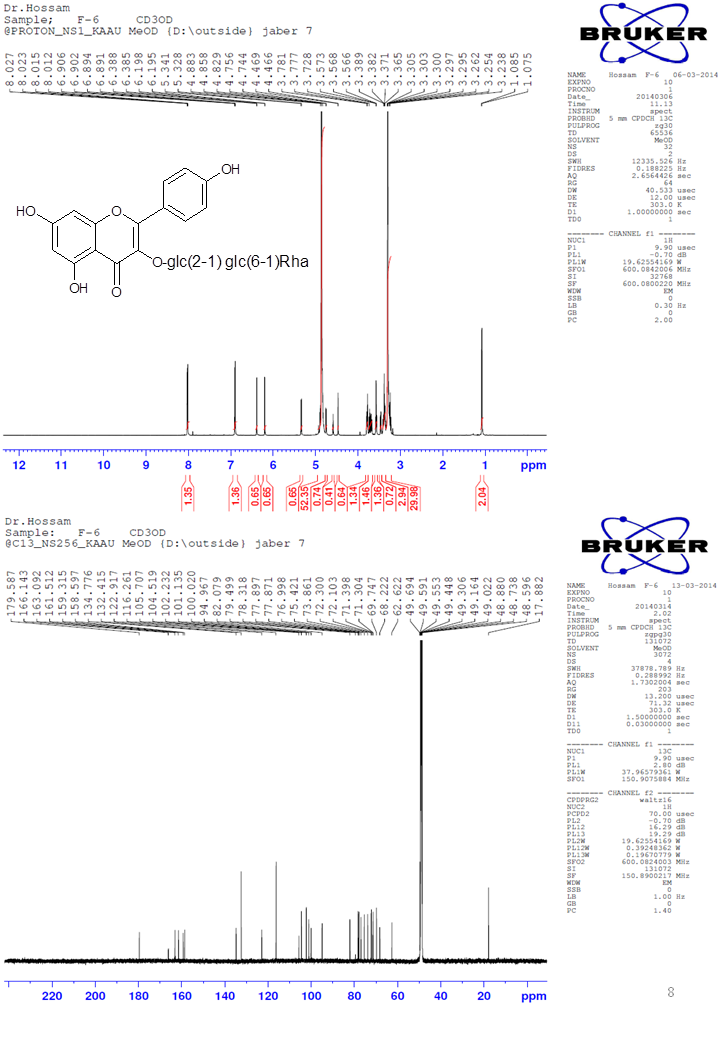

Supplement: Figure S5 — 1H-NMR and 13C-NMR charts of compound 6. (TIF) [file pone.0098559.s005.tif]

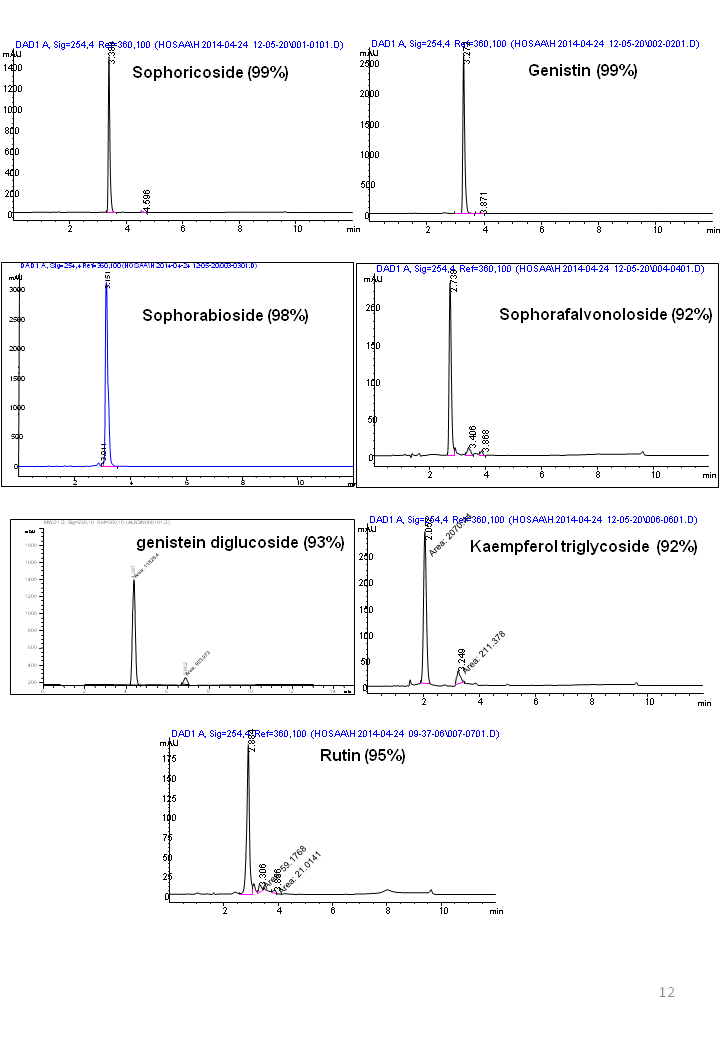

Supplement: Figure S6 — HPLC chromatograms of isolated compounds. (TIF) [file pone.0098559.s006.tif]
