# Supplementary material for: Isolation of Antiosteoporotic Compounds from Seeds of Sophora japonica
Source: PLoS One. 2014 Jun 3;9(6):e98559. doi: 10.1371/journal.pone.0098559 (PMC4043785; doi:10.1371/journal.pone.0098559)
Supplement: Supplementary file 2 [file pone.0098559.s007.docx]

^{13}C-NMR (200 MHz, MeOD):

	3	4	5	6
2	155.1	158.6	155.0	158.6
3	126.3	134.9	123.9	134.8
4	182.0	179.7	180.4	179.6
5	163.9	163.2	161.6	163.1
6	100.3	99.9	100.2	100.0
7	166.2	165.9	163.1	166.1
8	94.9	94.7	94.6	94.9
9	158.8	158.9	157.2	159.3
10	106.3	105.8	106.1	105.7
1`	124.3	122.8	122.2	122.9
2`	131.4	132.4	130.1	132.4
3`	117.4	116.3	116.0	116.3
4`	159.7	161.6	157.3	161.5
5`	117.4	116.3	116.0	116.3
6`	131.4	132.4	130.1	132.4
1``	100.3	101.0	99.8	101.1
2``	78.1	82.5	73.2	82.1
3``	79.1	77.9	76.6	76.9
4``	72.3	71.3	69.7	68.2
5``	79.3	78.2	77.2	77.8
6``	62.5	62.6	60.6	62.6
1```	102.5	104.7	99.6	104.5
2```	72.3	75.6	73.0	73.9
3```	71.5	77.9	76.4	75.4
4```	73.9	71.1	69.6	68.7
5```	69.9	78.3	77.0	72.3
6```	18.0	62.5	60.6	64.7
1````				102.2
2````				71.4
3````				71.3
4````				72.1
5````				69.7
6````				17.8
